# Supplementary material for: Host Response of Human Epidermis to Methicillin-Resistant Staphylococcus aureus Biofilm Infection and Synthetic Antibiofilm Peptide Treatment
Source: Cells. 2022 Nov 1;11(21):3459. doi: 10.3390/cells11213459 (PMC9657051; doi:10.3390/cells11213459)
Supplement: Supplementary file 1 [file cells-11-03459-s001.zip › cells-1936999-supplementary.pdf]

Supplementary Tables

Table S1. Inflammatory gene expression relative to untreated control skins. RT-qPCR of thermally burned skin samples infected with MRSA biofilm with or without DJK-5 treatment. Statistical analysis comparing each condition (3–4 replicates) to untreated skin was performed using one-way ANOVA, Dunnett's multiple comparison test (* p ≤ 0.05; ** p ≤ 0.01; *** p ≤ 0.001; **** p ≤ 0.0001).

Gene	Fold change in gene expression (mean ± SD)				
	DJK-5 control	Burned skin	Burned skin + DJK-5	Burned skin + MRSA biofilm	Burned skin + MRSA biofilm + DJK-5
IL-8	1.1 ± 0.30	0.9 ± 0.28	1.1 ± 0.23	24.9 ± 2.33 ****	4.2 ± 3.50
IL-1β	1.0 ± 0.30	1.2 ± 0.12	2.1 ± 0.32	53.7 ± 4.67 ****	10.8 ± 1.02 ****
IL-1α	1.2 ± 0.17	1.0 ± 0.48	1.5 ± 0.26	20.3 ± 1.69 ****	3.8 ± 2.75 *
IL-6	0.7 ± 0.12	0.7 ± 0.13	1.0 ± 0.18	1.6 ± 0.26 **	1.3 ± 0.31

Table S2. Human RT-qPCR Primer Sequences used in Supplementary TABLE 1.

Gene	Forward	Reverse
IL-8	GCCAGGAAGAAACCACCGGAAGG	GGCTGCCAAGAGAGCCACGG
IL-1β	ACGAATCTCCGACCACCACT	CCATGGCCACAACAACTGAC
IL-1α	CGCCAATGACTCAGAGGAAGA	AGGGCGTCATTCAGGATGAA
IL-6	GGTACATCCTCGACGGCATCT	GTGCCTCTTTGCTGCTTTCAC
B2M	GATGAGTATGCCTGCCGTGTG	CAAACCTCGGGTAGCATCAT
RPLP0	CACCATTGAAATCCTGAGTGATGT	TGACCAGCCCAAAGGAGAAG

Table S3. DJK-5 Suppressed Inflammatory Cytokine and Chemokine Production from MRSA Biofilm Infected Burned Skin. ELISA of MRSA biofilm infected burned skin samples with or without DJK-5 treatment. Statistical analysis comparing DJK-5 treated biofilm to MRSA biofilm infected skin (15-18 replicates) was performed using unpaired t-test (* p ≤ 0.05; ** p ≤ 0.01; *** p ≤ 0.001; **** p ≤ 0.0001).

Cytokine	Cytokine levels quantified from skin culture medium (mean ± SD)		
	Burned skin + MRSA biofilm (pg/mL)	Burned skin + MRSA biofilm + DJK-5 (pg/mL)	Burned skin control (pg/mL)
IL-8	3796.2 ± 1368.9	1437.6 ± 897.7 ****	472.9 ± 158.1
IL-1β	665.5 ± 216.3	77.2 ± 33.8 ****	2.0 ± 1.7

Table S4. Differential Gene Expression of Representative Genes in Different Biological Functions in RNA-Seq Analysis.

Function	Comparison	Burned skin + MRSA biofilm vs. Burn skin		Burned skin + MRSA biofilm + DJK-5 vs. Burned skin + MRSA biofilm	
	Gene	Fold change	Adjusted p-value	Fold change	Adjusted p-value
Inflammatory cytokines	IL-17C	803.80	2.62×10^{-12}	−24.0	2.16×10^{-4}
	IL-23A	68.30	6.42×10^{-14}	−6.2	1.47×10^{-3}
Keratins	KRT1	−4.95	5.13×10^{-6}	3.67	2.58×10^{-4}
	KRT2	−18.43	2.73×10^{-9}	9.23	7.77×10^{-6}
	KRT9	−5.36	5.22×10^{-11}	2.50	5.83×10^{-4}
	KRT10	−8.63	3.09×10^{-8}	5.48	1.65×10^{-5}
ECM proteins	LAMA1	5.97	4.45×10^{-31}	−3.75	2.48×10^{-17}
	LAMA3	5.70	6.67×10^{-18}	−3.15	2.11×10^{-8}
	ITGA2	7.22	3.16×10^{-18}	−3.48	6.35×10^{-8}
	ITGA5	14.31	2.22×10^{-32}	−7.41	1.08×10^{-18}
	ITGB4	6.60	1.01×10^{-36}	−5.00	7.89×10^{-27}
	MMP1	3.90	3.92×10^{-8}	−1.74	3.05×10^{-2}
	MMP3	12.63	1.05×10^{-14}	−11.05	4.85×10^{-13}
	MMP9	5.01	1.04×10^{-10}	−2.70	9.61×10^{-5}

DNA repair	BRCA1	−2.55	4.12×10^{-5}	3.11	8.43×10^{-7}
	BRCA2	−4.93	2.71×10^{-5}	7.12	2.75×10^{-7}
	RFC2	−2.71	1.11×10^{-14}	2.56	6.34×10^{-13}
	RFC3	−2.49	6.30×10^{-5}	3.56	2.74×10^{-8}

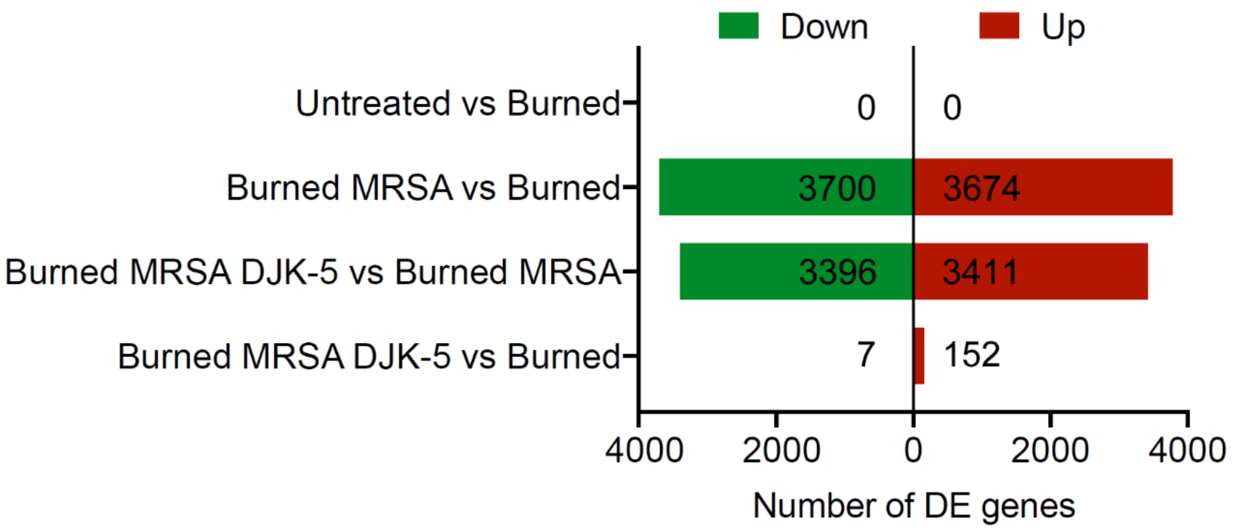

Figure S1. Differential expression analysis of MRSA biofilm infected thermally-injured skin treated with topical DJK-5. One-day MRSA USA300-LAC skin biofilms associated with thermally-damaged N/TERT epidermal skin were treated with vehicle control (water) or DJK-5 peptide at a dosage of 0.4% (120 µg) for 24 h. RNA-Seq libraries were sequenced on a HiSeqX sequencer (Illumina). Differential expression analysis was performed using DESeq2 v1.28.1, and significance was determined based on adjusted p-value ≤0.05 and fold change ≥ ±1.5.
